# Supplementary material for: Risk of nontyphoidal Salmonella bacteraemia in African children is modified by STAT4
Source: Nat Commun. 2018 Mar 9;9:1014. doi: 10.1038/s41467-017-02398-z (PMC5844948; doi:10.1038/s41467-017-02398-z)
Supplement: Supplementary file 1 — Supplementary Information [file 41467_2017_2398_MOESM1_ESM.pdf]

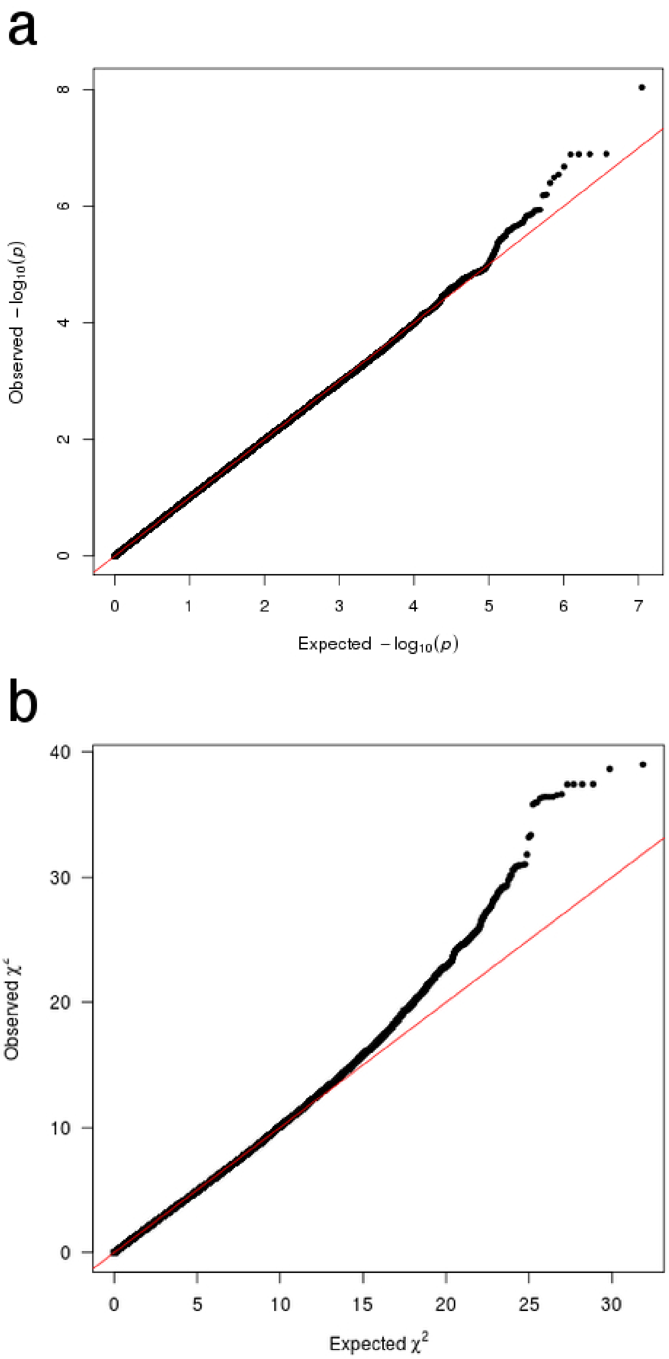

Supplementary Figure 1 | Quantile-quantile plots of NTS association in Kenyan discovery samples. Under (**a**) additive (5,585,198 SNPs, λ = 1.01) and (**b**) genotypic (4,669,480 SNPs, λ = 1.01) models. Observed -$\log_{10}$(P-values)/test statistics are plotted against those expected under a χ^2 distribution with 1 degree of freedom (additive model) and two degrees of freedom (genotypic model). For reference, genome-wide inflation factors under recessive dominant and heterozygous advantage models of association are 1.02, 1.00, and 1.00 respectively.

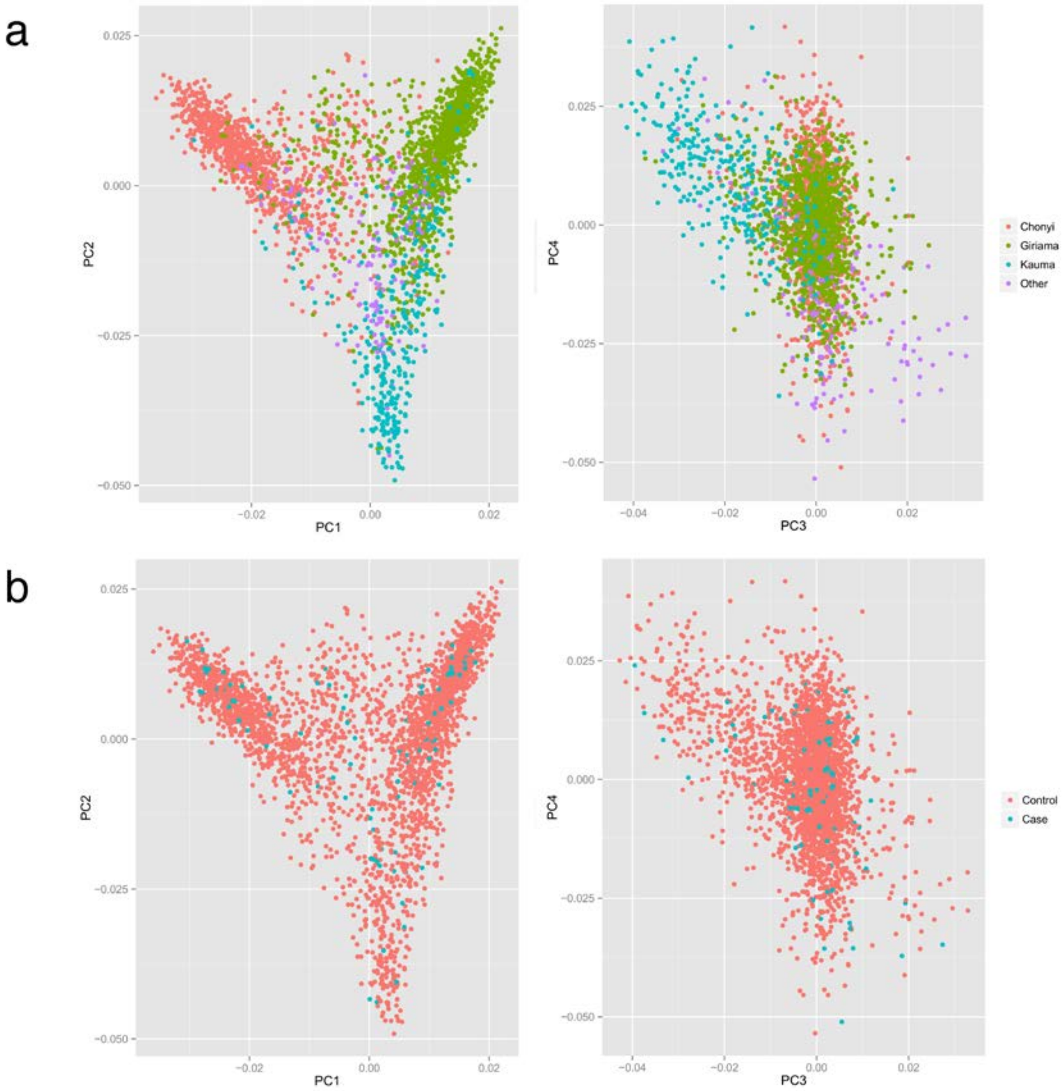

Supplementary Figure 2 | Principal components of Kenyan discovery genome-wide genotyping data. Individuals are color-coded according to (**a**) self-reported ethnicity and (**b**) case-control status. The first four principal components are displayed.

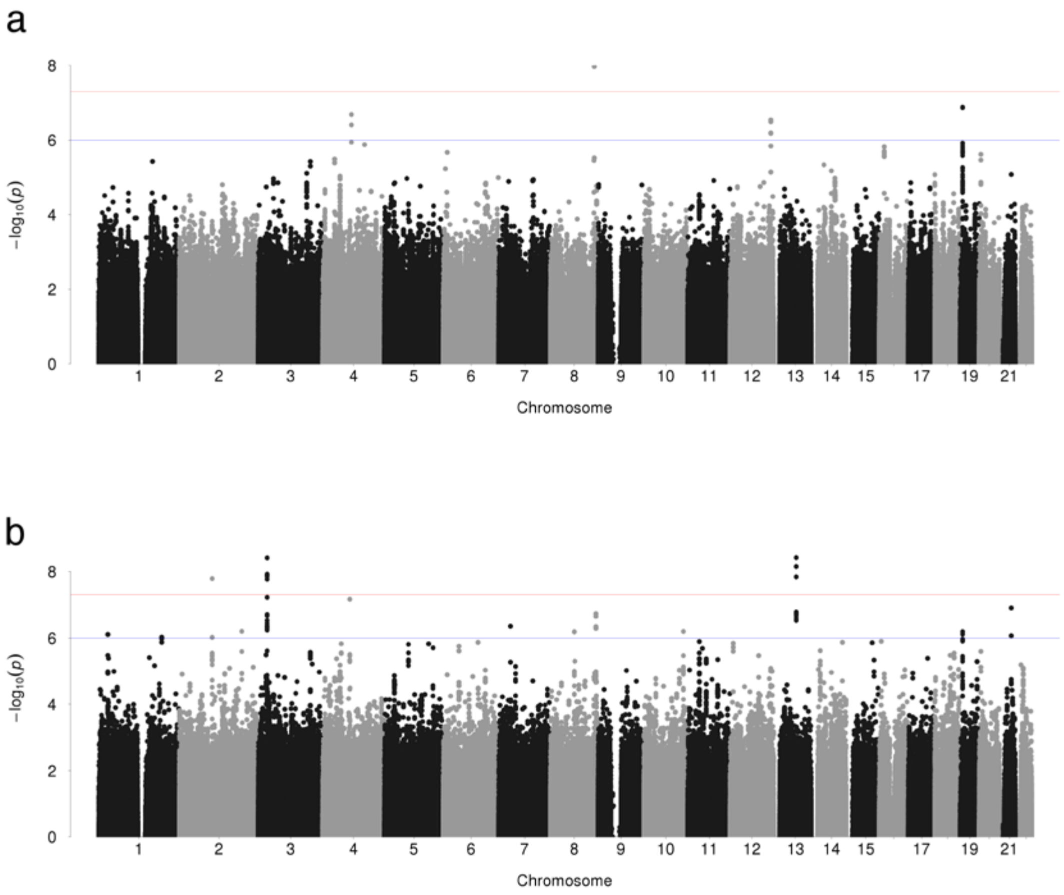

Supplementary Figure 3 | Manhattan plots of NTS association in Kenyan discovery samples. Under (**a**) additive (5,585,198 SNPs) and (**b**) genotypic (4,669,480 SNPs) models of association. The upper (red) horizontal line denotes genome-wide significance (P=5x10^{-8}), the lower (blue) horizontal denotes suggestive association (P=1x10^{-6}) used as the threshold for replication.

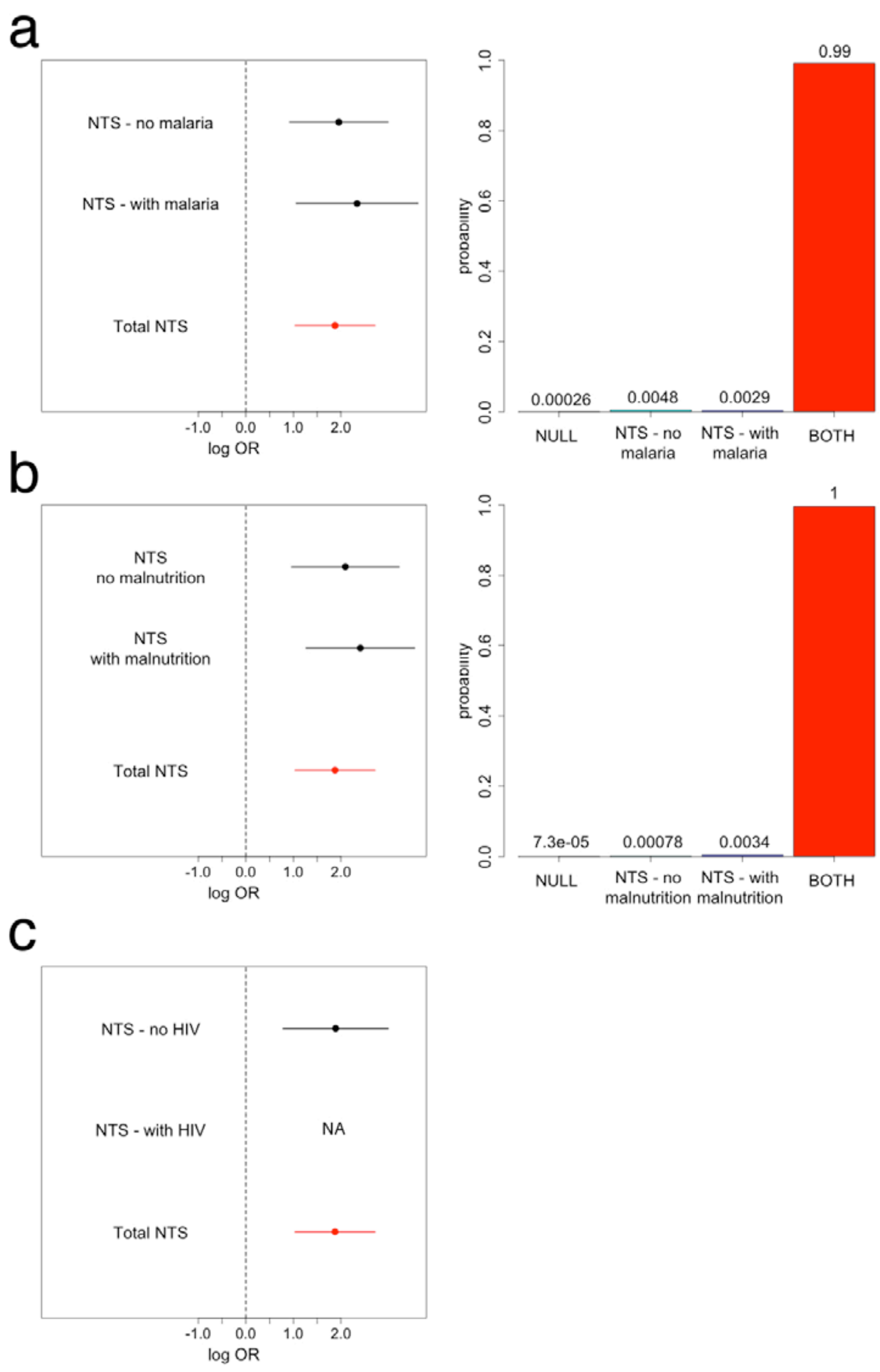

Supplementary Figure 4 | rs13390936 association with NTS bacteraemia in Kenyan children with and without acquired risk factors for NTS. Log-transformed odds ratios and 95% confidence intervals of rs13390936 association (recessive model) with NTS bacteraemia in Kenyan discovery samples (left panels), stratified by (**a**) malaria co-infection, (**b**) malnutrition (severe wasting*), and (**c**) HIV co-infection among cases. Posterior probabilities of models of association at rs13390936 (right panels): NULL, no association with any NTS serovar; NTS – no malaria/malnutrion, a non-zero effect in NTS bacteraemia without malaria/malnutrion alone; NTS – with malaria/malnutrion, a non-zero effect in NTS bacteraemia with malaria/malnutrion alone;

BOTH, the same non-zero effect in both children with and without acquired risk factors. The models in which NTS bacteraemia in children with and without malaria and malnutrition (highlighted in red) is associated with rs13390936 with the same effect size are the most probable (Bayes factor c.f. NULL = 3,784 and 13,583 respectively). The number of HIV-infected children with NTS bacteraemia (n=24) in the Kenyan discovery samples is too small to permit a stratified analysis.

*Severe wasting defined as weight-for-age z-scores <-3.

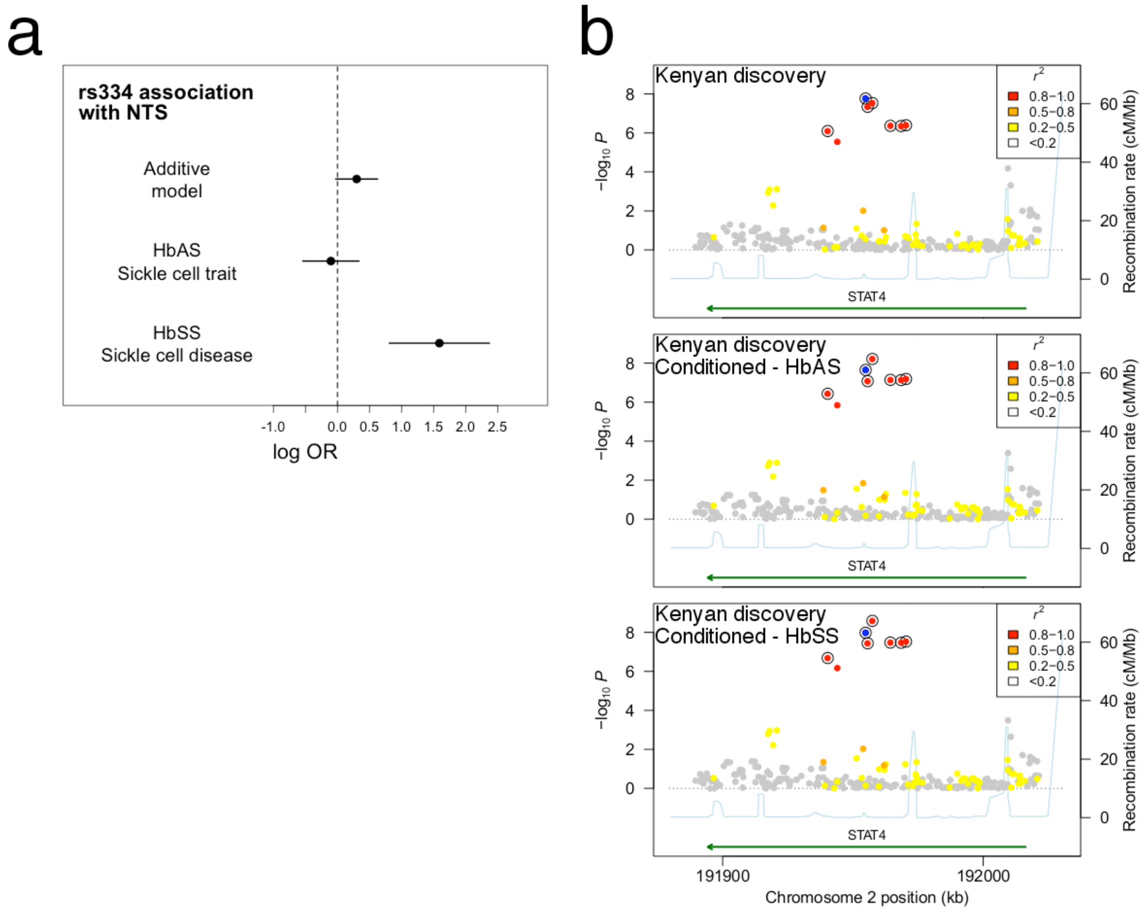

Supplementary Figure 5 | Association at the sickle cell locus (rs334) with NTS bacteraemia in the Kenyan discovery samples (a). Genotype at rs334 (cases=164, HbAA=129, HbAS=26, HbSS=9; controls=2,342, HbAA=1,876, HbAS=437, HbSS=29) is not associated with risk of NTS bacteraemia in Kenyan children under an additive model (logistic regression, *P*=0.07; OR 1.35 95% CI 0.96-1.85). There is no statistically significant evidence for a protective effect of HbAS carriage (HbAS vs. HbAA) on risk of NTS bacteraemia in these children (logistic regression, *P*=0.65; OR 0.90 95% CI 0.57-1.38). Carriage of HbSS (HbSS vs. HbAA) is associated with increased risk of NTS bacteraemia (logistic regression, P=8.30x10^{-5}; OR 4.89 95% CI 2.10-10.40). **Association at the STAT4 locus with NTS bacteraemia is independent of genotype at the sickle cell locus (b).** NTS-association at the *STAT4* region under the recessive model not conditioned on genotype (top panel) in Kenyan discovery samples (n=180 cases, 2,677 controls). rs13390936 is highlighted in blue. SNPs are coloured according to strength of linkage disequilibrium (r^2) to rs13390936. SNPs in a credible SNP set, which includes the causal variant with >95% probability, are ringed with black circles. NTS association at the *STAT4* region is conditioned on genotype at rs334 under a heterozygote advantage model (HbAS carriage – middle panel) and under a recessive model (HbSS carriage – bottom panel).

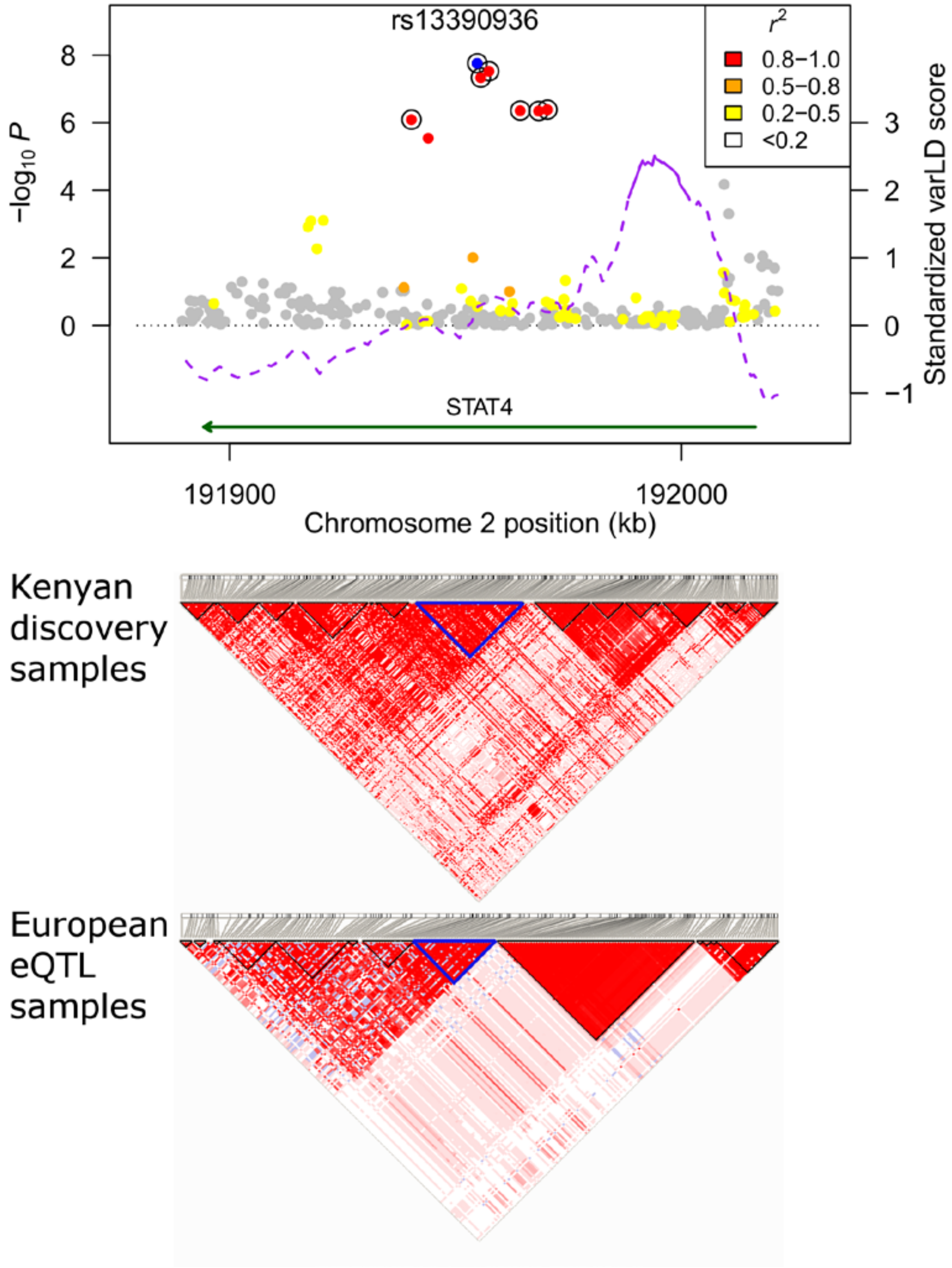

Supplementary Figure 6 | Linkage disequilibrium at the STAT4 locus in Kenyan and European study subjects. NTS association under a recessive model at the STAT4 locus (**top**) in Kenyan discovery samples (n = 180 cases, 2,677 controls). SNPs are colored according to strength of linkage disequilibrium (LD; r^2) to rs13390936 (in blue). SNPs in a credible SNP set, which includes the causal variant with >95% probability, are ringed with black circles. The plot is annotated with standardized varLD scores (purple line), quantifying the evidence for differential linkage disequilibrium between Kenyan discovery samples and European samples used in the eQTL analyses (n=421). The dashed purple line indicates regions with no evidence for differential LD between the two populations, the solid purple line indicates evidence of differential LD (varLD score >95th centile). LD plots (Haploview v4.2) of the STAT4 region in the Kenyan (**middle**) and European (**bottom**) samples, have the haplotype containing rs13390936 highlighted with a blue triangle. Red squares indicate strong evidence of pairwise LD (*D’*=1, LOD score – log odds of data likelihood under LD vs linkage

equilibrium - >2); pink squares indicate LOD>2, *D’*<1; blue squares, *D’*=1, LOD<2; white squares, *D’*<1, LOD<2.

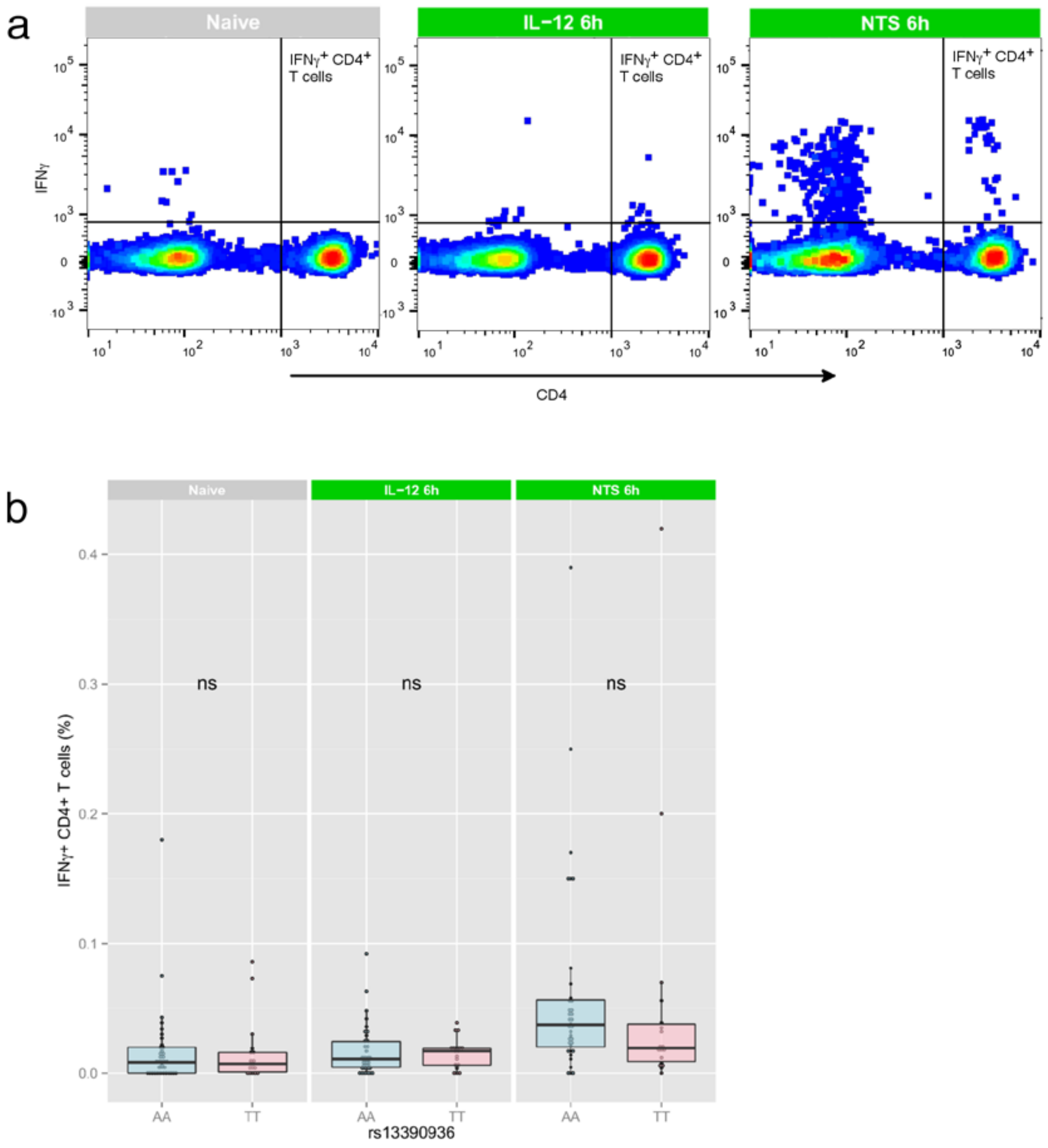

Supplementary Figure 7 | Effect of rs13390936 genotype on IFNγ production in CD4$^+$ T cells. (**a**) Gating strategy for IFNγ$^+$ CD4$^+$ T cells. Representative IFNγ production in naïve, IL-12- and NTS-stimulated CD4$^+$ T cells. (**b**) The proportion of IFNγ$^+$ CD4$^+$ T cells is not modified by rs13390936 genotype in naïve ($mean_{AA}$ = 0.017%, $mean_{TT}$ = 0.016%, Wilcoxon rank-sum P = 0.941), IL-12 stimulated ($mean_{AA}$ = 0.017%, $mean_{TT}$ = 0.015%, Wilcoxon rank-sum P = 0.727) or NTS-stimulated ($mean_{AA}$ = 0.062%, $mean_{TT}$ = 0.055%, Wilcoxon rank-sum P = 0.132) cells in 54 (protective AA genotype, n = 36; susceptible TT genotype, n = 18) healthy European adults. ns, not significant.

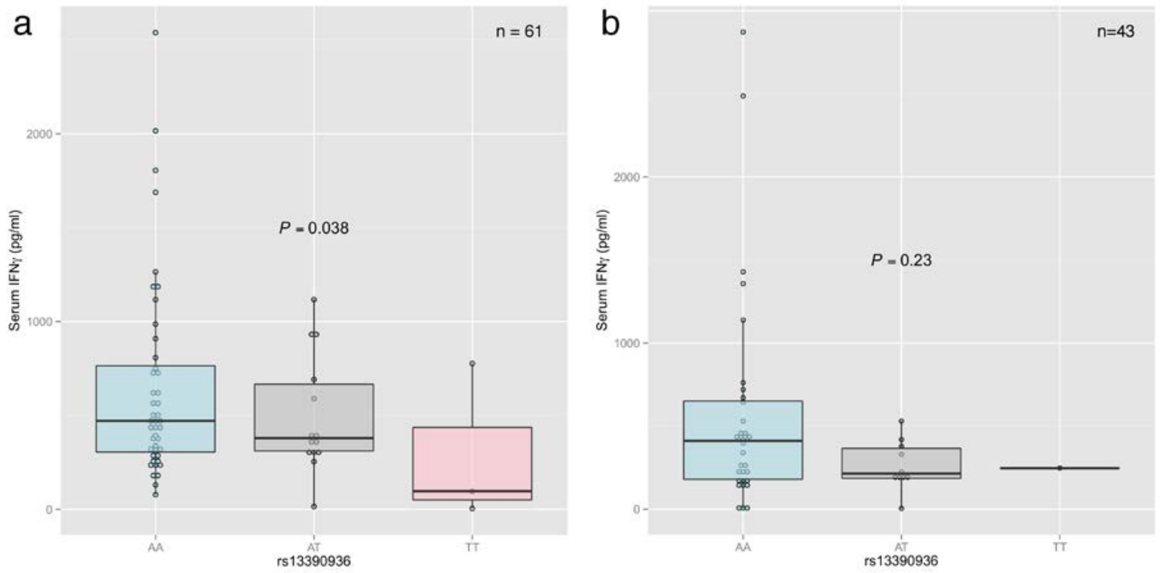

Supplementary Figure 8 | IFNγ production in HIV-infected and HIV-uninfected Malawian children with NTS bacteraemia. (**a**) HIV-uninfected Malawian children (total n=61; rs13390936:AA, n=44; TA, n=14; TT, n=3) with the NTS-susceptibility (T) allele have reduced circulating levels of IFNγ during acute NTS bacteraemia ($mean_{AA}$ = 650.3pg/ml, $mean_{TT}$ = 292.6pg/ml), in a linear model adjusted for age, sex, malaria and malnutrition. (**b**) HIV-infected Malawian children (total n=43; rs13390936:AA, n=32; TA, n=10; TT, n=1) with the NTS-susceptibility (T) allele do not have statistically significantly reduced circulating levels of IFNγ during acute NTS bacteraemia ($mean_{AA}$ = 568.9pg/ml, $mean_{TT}$ = 247.7pg/ml), in a linear model adjusted for age, sex, malaria and malnutrition. P-values are calculated by linear regression and ANOVA.

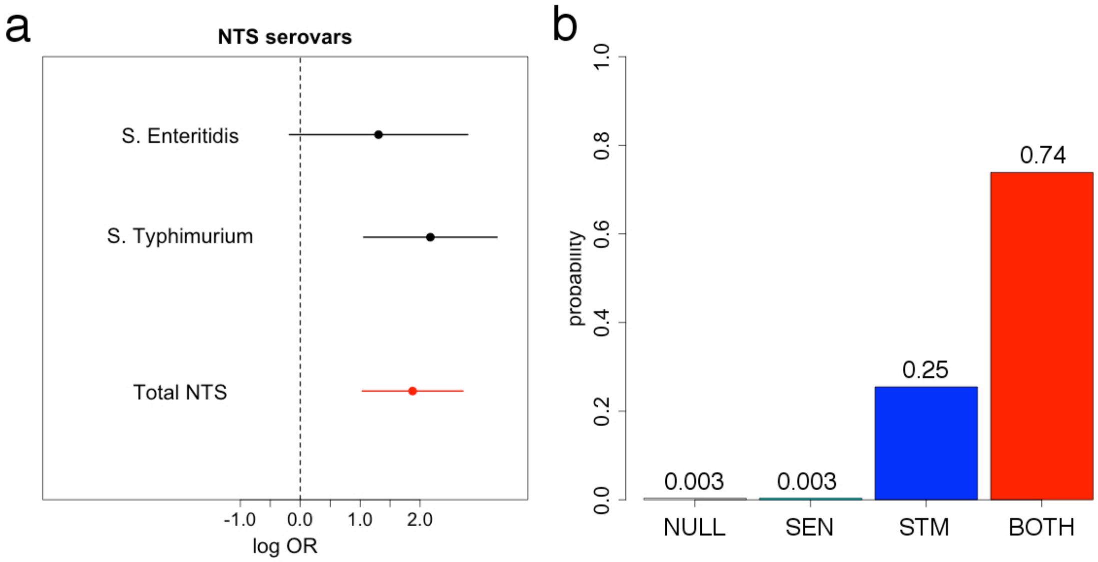

Supplementary Figure 9 | (a) rs13390936 association with major NTS serovars in Kenyan children. Log-transformed odds ratios and 95% confidence intervals of rs13390936 association (recessive model) in Kenyan discovery samples. (**b**) Posterior probabilities of models of association at rs13390936: NULL, no association with any NTS serovar; SEN, a non-zero effect in *S*. Enteritidis bacteraemia alone; STM, a non-zero effect in *S*. Typhimurium bacteraemia alone; BOTH, the same non-zero effect in both NTS serovars. In a comparison of all possible combinations of rs13390936 association with NTS serovars, the model in which bacteraemia secondary to both *S*. Typhimurium and *S*. Enteritidis (highlighted in red) is associated with rs13390936 is the most probable (Bayes factor c.f. NULL = 225).

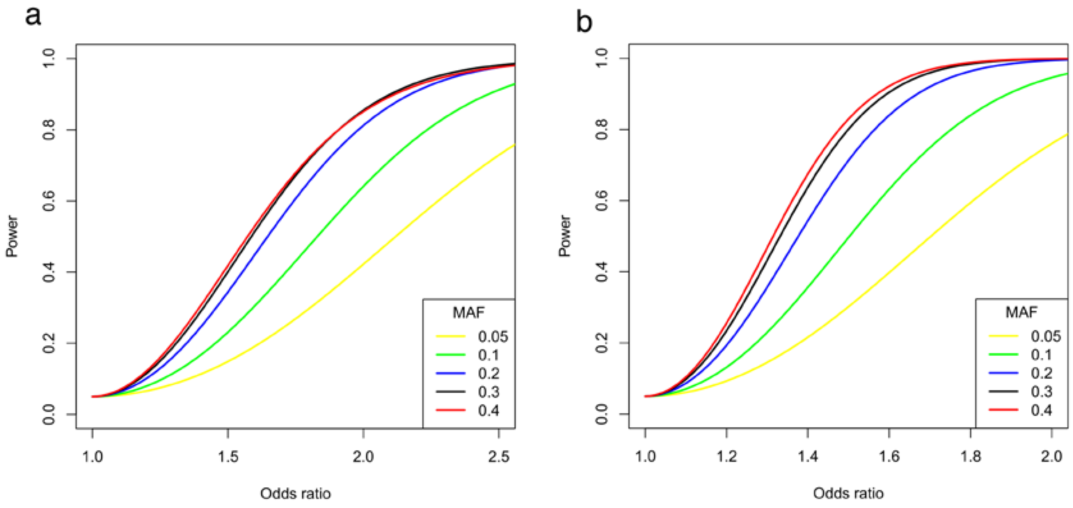

Supplementary Figure 10 | Statistical power in replication experiments. Statistical power to detect an association ($P<0.05$) of varying effect size in (**a**) Kenyan replication samples (38 cases, 1,336 controls) and (**b**) Malawian replication samples (150 cases, 339 controls) given different minor allele frequencies (MAF).

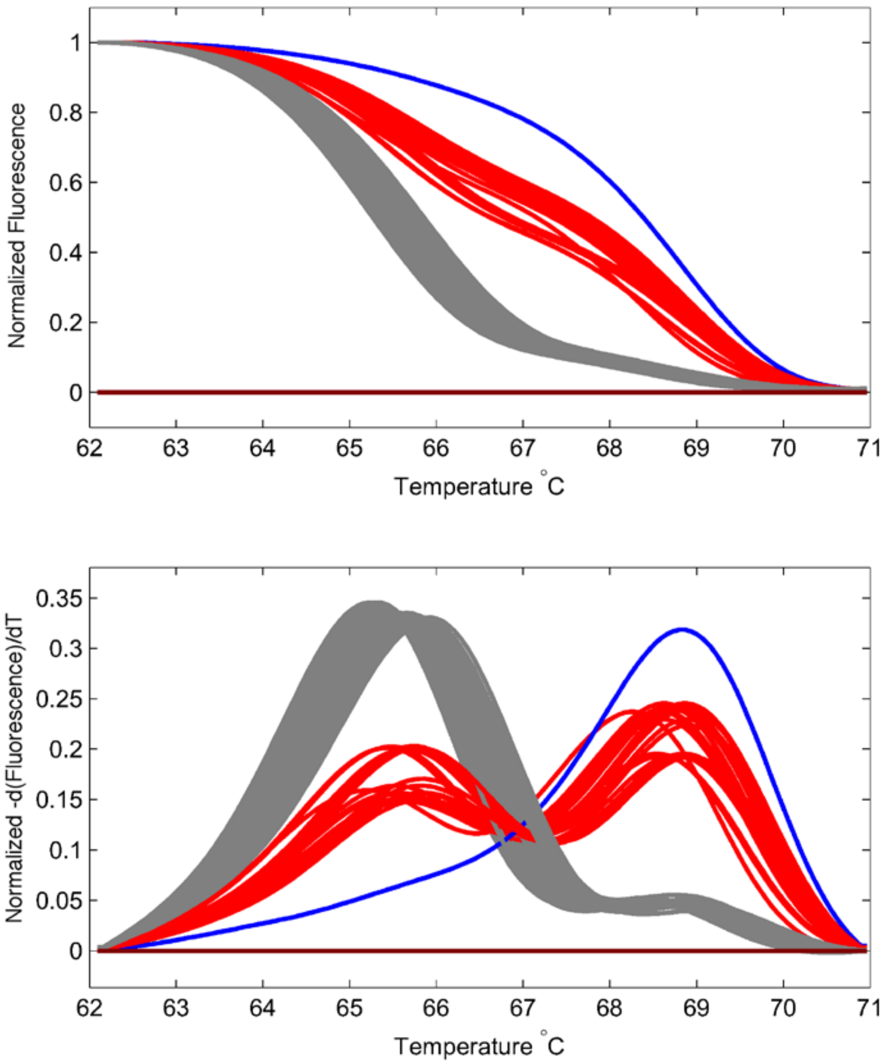

Supplementary Figure 11 | HRMA genotyping of rs13390936. Representative normalized melting curves (**top**) and peaks (**bottom**) from rs13390936 HRMA genotyping. Data is taken from a single assay (96 individuals). Genotypes are color-coded as follows; AA, grey, AT red, TT blue.

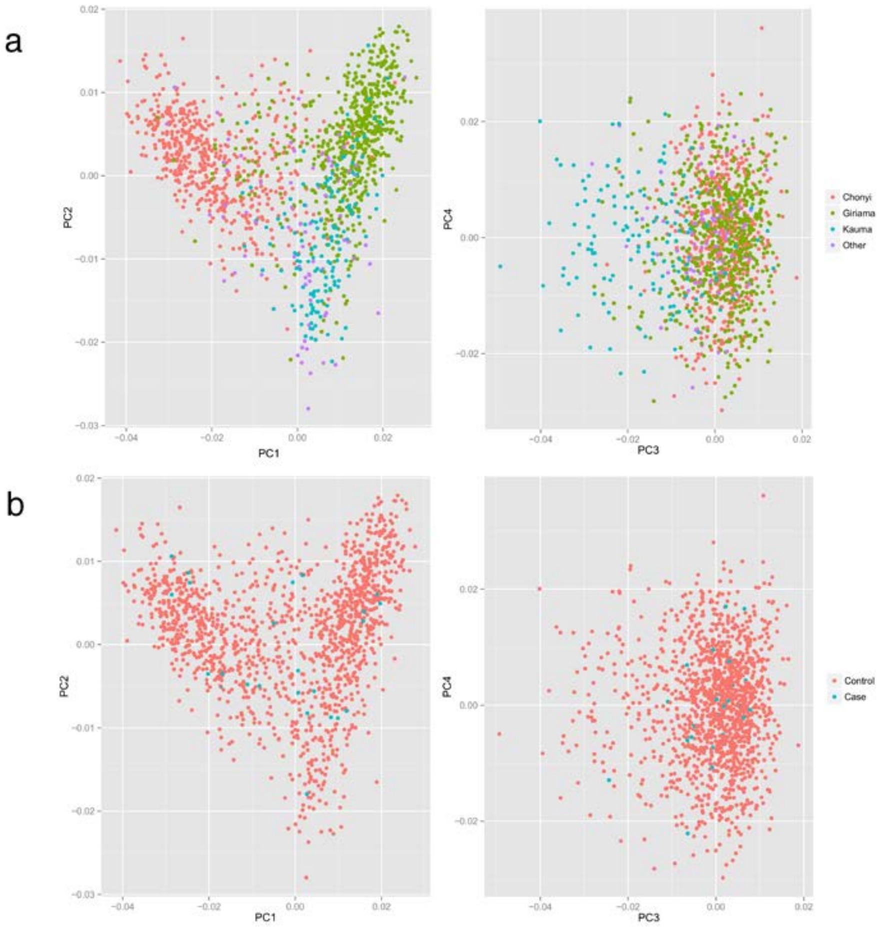

Supplementary Figure 12 | Principal components of Kenyan replication genome-wide genotyping data. Individuals are color-coded according to (**a**) self-reported ethnicity and (**b**) case-control status. The first four principal components are displayed.

		Kenyan discovery samples		Kenyan replication samples	
		Cases	Controls	Cases	Controls
	Total before QC	218	3,000	49	1,444
Exclusion criteria	Call rate & extreme heterozygosity	7	99	6	40
	HapMap PCA outliers	15	68	2	18
	Outlying channel intensity	0	12	1	1
	Sequenom discordance	0	13	0	8
	Discrepant or undetermined gender	4	14	1	2
	Duplicate samples	4	16	2	3
	Relatedness (IBD>0.4)	1	83	0	28
	Duplication of discovery samples in replication	NA	NA	1	12
	Genotyping failures	8	37	0	0
	Total after QC	180	2,677	38	1,336

Supplementary Table 1 | Kenyan discovery & replication sample exclusions. Sample exclusions in the Affymetrix SNP 6.0 chip-genotyped Kenyan discovery and ImmunoChip-genotyped Kenyan replication sets. QC, quality control; IBD, Identity by descent.

Genotyped SNP exclusions	Minor allele frequency <1%	50,322	
	Info <0.975	53,419	
	HWE P<1x10^{-20}	18,288	
	Plate effect P<1x10^{-6}	7,382	
	SNP missingness >2%	34,430	
	**Genotyped, autosomal SNPs taken forward for imputation**	**787,861**	
		Additive model	Genotypic model
Imputed SNP exclusions	HWE *P*<1x10^{-10}	1,684	
	Minor allele frequency <10%	8,774,454	
	Imputation info <0.8*	572,862	1,439,881
	**Imputed, autosomal SNPs included in analysis**	**5,585,198**	**4,669,480**

Supplementary Table 2 | Kenyan discovery GWAS SNP exclusions. SNP exclusions in the Affymetrix SNP 6.0 chip-genotyped Kenyan discovery samples, pre- and post-imputation. HWE, Hardy Weinberg Equilibrium.

*While the imputation info threshold (0.8) is used in both additive and genotypic analyses, the metric is model specific, resulting in larger numbers of SNP exclusions in the genotypic analysis.

SNP				NTS association			r^2 to peak SNP in locus
CHR	BP (b37)	RSID	Freq.	Info Score	*P*	Model	
1	29053720	rs4654369	0.264	0.969	7.88E-07	Genotypic	1.000
1	29054752	rs10799120	0.264	0.971	7.85E-07	Genotypic	1
1	194946012	rs866059	0.115	0.976	9.46E-07	Genotypic	1
2	100773069	rs184422605	0.190	0.961	9.64E-07	Genotypic	0.770
2	100789798	rs75776491	0.232	0.988	9.56E-07	Genotypic	0.978
2	100795043	rs62150289	0.238	0.923	1.63E-08	Genotypic	1
2	191954816	rs13390936	0.124	0.878	6.33E-07	Genotypic	1
3	26761622	rs1488241	0.147	0.995	3.68E-07	Genotypic	0.780
3	26768800	rs2171512	0.135	1.000	4.75E-07	Genotypic	0.730
3	26768818	rs2129855	0.135	1.000	5.83E-07	Genotypic	0.727
3	26769354	rs11713454	0.135	0.999	4.63E-07	Genotypic	0.730
3	26769392	rs7430878	0.135	0.999	4.63E-07	Genotypic	0.730
3	26769429	rs6790025	0.135	0.999	4.63E-07	Genotypic	0.730
3	26771574	rs139655208	0.135	0.995	4.35E-07	Genotypic	0.730
3	26792607	rs11914329	0.145	0.951	1.31E-08	Genotypic	0.803
3	26792972	rs961399	0.145	0.951	1.31E-08	Genotypic	0.803
3	26793518	rs1488248	0.145	0.953	1.39E-08	Genotypic	0.803
3	26794821	rs1488250	0.136	1.000	5.28E-07	Genotypic	0.736
3	26795026	rs1505577	0.145	1.000	6.04E-08	Genotypic	0.817
3	26795765	rs1488251	0.135	0.999	3.00E-07	Genotypic	0.751
3	26796445	rs6768401	0.134	1.000	2.00E-07	Genotypic	0.752
3	26796598	rs1586506	0.134	0.999	2.00E-07	Genotypic	0.753
3	26796765	rs1586507	0.134	0.999	1.96E-07	Genotypic	0.752
3	26798069	rs1488254	0.143	0.985	1.70E-08	Genotypic	0.819
3	26798614	rs1032885	0.142	0.979	1.25E-08	Genotypic	0.814
3	26798992	rs4973809	0.142	0.978	1.20E-08	Genotypic	0.814
3	26799072	rs6778280	0.143	0.978	1.35E-08	Genotypic	0.820
3	26799395	rs1038636	0.143	0.977	1.33E-08	Genotypic	0.820
3	26800829	rs6551141	0.157	0.848	2.12E-07	Genotypic	0.801
3	26804954	rs10865806	0.158	0.932	3.85E-09	Genotypic	1
4	83324569	rs11099533	0.111	0.858	6.86E-08	Genotypic	1
4	88413965	rs17012693	0.214	0.920	2.06E-07	Additive	1
4	88420287	rs2169502	0.222	0.923	3.95E-07	Additive	0.939
7	35394619	rs138302119	0.115	0.848	4.47E-07	Genotypic	1
8	73087935	rs1809447	0.113	0.825	6.61E-07	Genotypic	1.000
8	73090061	rs13251057	0.113	0.822	6.49E-07	Genotypic	1
8	134298504	rs16904882	0.112	0.954	1.08E-08	Additive	1
8	139215025	rs11995943	0.184	0.836	5.20E-07	Genotypic	0.980
8	139215155	rs11985359	0.184	0.831	5.05E-07	Genotypic	0.980
8	139215247	rs13265099	0.184	0.826	4.60E-07	Genotypic	0.983
8	139215574	rs4404965	0.189	0.806	1.85E-07	Genotypic	1
8	139215649	rs7387858	0.188	0.806	2.23E-07	Genotypic	0.993
10	121497290	rs73355807	0.154	0.915	6.40E-07	Genotypic	1
12	125613150	rs78857905	0.123	0.832	2.89E-07	Additive	1
12	125614614	rs76336213	0.118	0.806	6.65E-07	Additive	0.998
12	125615610	rs75239828	0.118	0.805	6.44E-07	Additive	0.998
12	125615627	rs75627386	0.122	0.804	3.26E-07	Additive	0.964
13	69573095	rs73211763	0.112	0.830	2.99E-07	Genotypic	0.901
13	69576658	rs73211791	0.112	0.836	2.56E-07	Genotypic	0.901
13	69578250	rs73213609	0.112	0.832	2.10E-07	Genotypic	0.901
13	69579168	rs55763812	0.126	0.999	1.45E-08	Genotypic	0.998
13	69579594	rs73213613	0.124	1.000	7.10E-09	Genotypic	1.000
13	69580147	rs1340723	0.112	0.827	1.80E-07	Genotypic	0.901
13	69580255	rs9541701	0.124	1.000	7.05E-09	Genotypic	1.000
13	69580286	rs9541702	0.124	1.000	7.04E-09	Genotypic	1.000
13	69581350	rs7321066	0.112	0.824	1.65E-07	Genotypic	0.901
13	69581690	rs9541704	0.124	1.000	6.99E-09	Genotypic	1.000
13	69582697	rs12429197	0.111	0.811	1.87E-07	Genotypic	0.899
13	69582740	rs9541709	0.124	0.955	3.78E-09	Genotypic	1
19	7914568	rs550134	0.349	0.908	1.32E-07	Additive	1
19	7915451	rs520802	0.440	0.847	6.52E-07	Genotypic	0.720
19	7916475	rs2452012	0.349	0.908	1.34E-07	Additive	1.000
19	7916620	rs580790	0.349	0.908	1.33E-07	Additive	1.000
19	7922672	rs2059820	0.348	0.907	1.35E-07	Additive	1.000
19	7932459	rs523648	0.432	0.834	7.70E-07	Genotypic	0.740
21	35289246	rs2834301	0.158	1.000	8.59E-07	Genotypic	0.422
21	35303773	rs8129054	0.275	0.805	1.25E-07	Genotypic	1

Supplementary Table 3 | Association between NTS bacteraemia and genotype at suggestively associated SNPs. SNP (n = 67) suggestively associated with NTS bacteraemia ($P<1x10^{-6}$) at 16 loci in Kenyan discovery

samples are displayed. CHR, chromosome; BP, base-pair position (Human Genome Reference GRCh37); RSID, SNP identifier; Freq, minor allele frequency.

				Kenyan discovery samples (180 cases; 2,677 controls)					Kenyan replication samples (36 cases; 282 controls)				Malawian replication samples (135 cases; 281 controls)				Fixed effects meta-analysis		
CHR	BP (b37)	SNP	Gene	MAF cases imputed	MAF controls imputed	Imputation accuracy	*P* imputed	OR (95% CI) imputed	MAF cases	MAF controls	*P*	OR (95% CI)	MAF cases	MAF controls	*P*	OR (95% CI)	*P*	OR (95% CI)	Model
1	29053720	rs4654369		0.32	0.26	0.97	7.88x10^{-7}		0.22	0.27	0.436		0.29	0.28	0.981				Genotypic
1	194946012	rs866059		0.09	0.12	NA	9.46x10^{-7}		NA	NA	NA		NA	NA	NA				Genotypic
2	100795043	rs62150289	*AFF3***	0.32	0.23	0.95	1.63x10^{-8}		0.18	0.21	0.588		0.18	0.22	0.471				Genotypic
2	191940260	rs16833239	*STAT4***	0.17	0.13	0.95	1.80x10^{-5}		0.13	0.11	2.51x10^{-3}		0.15	0.13	0.080				Genotypic
**2**	**191954816**	**rs13390936**	***STAT4***	**0.17**	**0.12**	**0.97**	**6.33x10^{-7}**		**0.15**	**0.11**	**1.83x10^{-3}**		**0.15**	**0.14**	**0.039**				**Genotypic**
2	191968420	rs13407419	*STAT4***	0.17	0.13	0.97	1.18x10^{-5}		0.16	0.12	0.014		NA	NA	NA				Genotypic
2	191970330	rs13401064	*STAT4***	0.17	0.13	0.97	1.16x10^{-5}		0.13	0.11	4.03x10^{-3}		0.15	0.13	0.072				Genotypic
3	26768818	rs2129855	*LRRC3B**	0.17	0.13	NA	5.82x10^{-7}		0.14	0.14	0.940		0.13	0.14	0.218				Genotypic
3	26769354	rs11713454	*LRRC3B*	0.17	0.13	1.00	4.63x10^{-7}		0.14	0.14	0.926		0.13	0.13	0.206				Genotypic
3	26792607	rs11914329	*LRRC3B*	0.19	0.14	0.99	1.31x10^{-8}		0.15	0.15	0.779		0.13	0.14	0.451				Genotypic
3	26794821	rs1488250	*LRRC3B*	0.17	0.13	1.00	5.28x10^{-7}		0.14	0.14	0.940		0.14	0.14	0.461				Genotypic
3	26796445	rs6768401	*LRRC3B**	0.17	0.13	NA	2.00x10^{-7}		0.14	0.14	0.922		0.13	0.14	0.490				Genotypic
3	26798614	rs1032885	*LRRC3B*	0.18	0.14	1.00	1.25x10^{-8}		0.15	0.15	0.848		0.13	0.14	0.210				Genotypic
4	83324569	rs11099533		0.15	0.11	NA	6.86x10^{-8}		NA	NA	NA		NA	NA	NA				Genotypic
4	88413965	rs17012693	*SPARCL1*	0.33	0.21	0.95	2.06x10^{-7}	2.04 (1.56-2.66)	0.21	0.24	0.787	0.88 (0.35-2.23)	0.21	0.21	0.936	0.98 (0.6-1.6)	1.14x10^{-5}	1.66 (1.32-2.08)	Additive
7	35394619	rs138302119		0.16	0.11	NA	4.47x10^{-7}		NA	NA	NA		NA	NA	NA				Genotypic
8	73087935	rs1809447		0.16	0.11	0.99	6.61x10^{-7}		0.15	0.10	0.328		0.06	0.07	0.733				Genotypic
8	134298504	rs16904882	*NDRG1*	0.20	0.11	0.95	1.08x10^{-8}	2.78 (1.95-3.94)	0.00	0.11	0.988	NA	0.14	0.11	0.295	1.28 (0.81-2.01)	1.96x10^{-7}	2.08 (1.58-2.74)	Additive
8	139215155	rs11985359	*FAM135B*	0.16	0.19	0.99	5.05x10^{-7}		0.19	0.22	0.580		0.18	0.18	0.527				Genotypic
10	121497290	rs73355807	*INPP5F*	0.21	0.15	0.99	6.40x10^{-7}		0.17	0.14	0.630		0.08	0.11	0.246				Genotypic
12	125614614	rs76336213	*AACS*	0.19	0.11	0.93	6.65x10^{-7}	2.56 (1.77-3.70)	0.15	0.12	0.395	1.34 (0.68-2.63)	0.09	0.08	0.632	1.14 (0.67-1.93)	5.44x10^{-5}	1.80 (1.35-2.39)	Additive
13	69576658	rs73211791		0.15	0.11	0.99	2.56x10^{-7}		0.11	0.12	0.972		0.11	0.15	0.137				Genotypic
13	69582740	rs9541709		0.16	0.12	0.99	3.78x10^{-9}		0.11	0.12	0.993		0.18	0.17	0.956				Genotypic
19	7900650	rs652710	*EVI5L*	0.24	0.37	0.94	1.21x10^{-6}	0.56 (0.45-0.71)	0.40	0.36	0.550	1.17 (0.7-1.95)	0.33	0.36	0.474	0.89 (0.66-1.21)	1.60x10^{-4}	0.71 (0.60-0.85)	Additive
19	7903484	rs604938	*EVI5L***	0.34	0.46	0.99	2.17x10^{-5}	0.62 (0.50-0.78)	0.46	0.45	0.416	1.19 (0.75-1.9)	0.44	0.42	0.589	1.08 (0.81-1.44)	0.016	0.81 (0.69-0.96)	Additive
19	7910854	rs794442	*EVI5L***	0.33	0.45	0.99	1.27x10^{-5}	0.62 (0.50-0.77)	0.67	0.45	0.629	1.11 (0.69-1.76)	0.42	0.41	0.754	1.05 (0.79-1.39)	4.27x10^{-3}	0.79 (0.67-0.93)	Additive
19	7916620	rs580790	*EVI5L*	0.22	0.36	0.87	1.33x10^{-7}	0.53 (0.42-0.67)	0.40	0.31	0.110	1.51 (0.91-2.49)	0.30	0.34	0.382	0.88 (0.65-1.18)	1.23x10^{-4}	0.71 (0.6-0.85)	Additive
19	7922672	rs2059820	*EVI5L*	0.22	0.36	0.87	1.35x10^{-7}	0.53 (0.42-0.67)	0.42	0.32	0.356	1.39 (0.69-2.81)	0.29	0.31	0.529	0.9 (0.64-1.26)	1.72x10^{-5}	0.67 (0.55-0.80)	Additive
21	35289246	rs2834301	*ATP5O**	0.24	0.15	NA	8.59x10^{-7}		0.10	0.17	0.202		0.17	0.13	0.366				Genotypic

Supplementary Table 4 | Replication results and imputation accuracy of top SNPs (*P*<1x10^{-6}) in NTS Kenyan discovery analysis. Genotype data was generated by imputation using the Affymetrix SNP 6.0 chip (Kenyan discovery samples) and Sequenom MASSArray (Kenyan replication and Malawian replication). Where ImmunoChip genotyping data was available (SNPs

highlighted**), these data are presented. rs13390936 (highlighted in bold) was genotyped by High-Resolution Melt-curve Analysis (HRMA). Sample numbers are as indicated in the table, with the exception of Immunochip genotyped SNPs (Kenyan replication, 38 cases, 1,336 controls) and rs13390936 (Kenyan replication, 38 cases, 1,336 controls; Malawian replication, 150 cases, 339 controls). Imputation accuracy (proportion of correctly imputed genotypes) is calculated for a subset of Sequenom genotyped Kenyan discovery samples (180 cases, 750 controls), SNPs directly genotyped in the discovery analysis are highlighted (*). Odds ratios are given for SNPs associated with NTS disease in the Kenyan discovery samples under an additive model. For SNPs associated with disease under a genotypic model odds ratios are not reported. CHR, chromosome; BP (b37), base-pair position Human Genome Reference GRCh37; MAF, minor allele frequency; OR, odds ratio; CI, confidence interval.

	Genetic model									
Population	Genotypic		Additive		**Recessive**		Dominant		Heterozygous advantage	
	P	AIC	*P*	AIC	***P***	**AIC**	*P*	AIC	*P*	AIC
Kenya (discovery)	1.76×10^{-5}	1351	0.012	1359	$\mathbf{3.33 \times 10^{-6}}$	**1349**	0.141	1363	0.782	1365
Kenya (replication)	1.83×10^{-3}	340	0.398	346	$\mathbf{6.54 \times 10^{-4}}$	**339**	0.869	347	0.209	345
Malawi	0.039	586	0.559	589	**0.026**	**584**	0.917	589	0.379	589

Supplementary Table 5 | Genetic model comparison of rs13390936 association with NTS. Comparison of the observed rs13390936 association with NTS bacteraemia under alternative genetic models. AIC, Akaike information criterion.

			Kenya discovery (imputed)			Kenya replication		Malawi replication		Combined analysis*	
SNP	Position (chr:bp)	r^2 to rs13390936	OR (95% CI)	*P*	Imputation accuracy	OR (95% CI)	*P*	OR (95% CI)	*P*	OR (95% CI)	*P*
rs13390936	2:191954816	1	6.59 (2.68-14.78)**	1.74×10^{-8}	0.97	9.96 (2.66-37.36)	6.54×10^{-4}	4.89 (1.21-19.83)	0.026	6.81 (3.67-12.63)**	1.21×10^{-9}
rs16833239	2:191940260	0.97	5.87 (2.54-12.43)	8.07×10^{-7}	0.95	9.14 (2.47-33.86)	9.24×10^{-4}	5.41 (1.04-28.24)	0.045	6.38 (3.48-11.70)	1.99×10^{-9}
rs13401064	2:191970330	0.96	5.41 (2.24-11.80)	4.10×10^{-7}	0.97	7.51 (2.06-27.39)	2.24×10^{-3}	1.73 (0.46-6.54)	0.421	4.61 (2.54-8.37)	5.20×10^{-7}
rs12693593	2:191964323	0.96	5.41 (2.24-11.80)	4.37×10^{-7}							
rs13407419	2:191968420	0.96	5.41 (2.24-11.80)	4.48×10^{-7}	0.97	6.15 (1.72-21.90)	5.10×10^{-3}	8.59 (0.95-77.57)	0.056	5.81 (3.07-10.97)	5.98×10^{-8}
rs9789710	2:191957413	0.93	5.51 (2.51-11.18)	2.99×10^{-8}							
rs143464908	2:191955597	0.92	7.90 (2.94-19.40)	4.62×10^{-8}							

Supplementary Table 6 | NTS association (recessive model) at a set of SNPs in the STAT4 region with a 95% probability of containing the causal variant. rs16833239, rs13401064 and rs13407419 were genotyped using the Immunochip (Kenya replication samples) and Sequenom MASSArray assays (Malawi replication samples). rs16833239, rs13401064 and rs13407419 was also genotyped in 33% of the Kenyan discovery samples (n=938) using Sequenom MASSArray to confirm imputation accuracy. rs13390936 was genotyped in Kenyan and Malawian replication samples using HRMA, and imputation accuracy confirmed in 100% of the Kenyan discovery samples.

*Fixed effects meta-analysis of Kenyan discovery (imputed), Kenyan replication (directly genotyped) and Malawian replication (directly genotyped) populations. OR, odds ratio; CI, confidence interval.

**Discrepancies between p-values and effect sizes displayed for Kenyan discovery samples displayed here, and those reported in Table 1, reflect the use of imputed genotypes (here) and directly assayed genotypes (Table1).

		Kenya discovery (n=180)	Kenya replication (n=38)	Kenya total (n=218)	Malawi replication (n=150)	*P*
	Age (months)	15 (0-143)	14 (0-52)	15 (0-143)	16 (0-180)	0.050
	Female	85/180 (47.2%)	19/38 (50.0%)	104/218 (47.7%)	68/150 (45.3%)	0.732
NTS serovar	Typhimurium	66/166 (39.8%)	12/33 (36.4%)	78/199 (39.2%)	135/150 (90.0%)	<0.0001
	Enteritidis	84/166 (50.6%)	20/33 (60.6%)	104/199 (52.3%)	12/150 (8.0%)	<0.0001
	Not typable*	16/166 (9.6%)	1/33 (3.0%)	17/199 (8.5%)	3/150 (2.0%)	0.018
Co-morbidities	HIV-infection	24/117 (20.5%)	6/30 (20.0%)	30/147 (20.4%)	49/126 (38.9%)	0.001
	Malaria	55/179 (30.7%)	15/38 (39.5%)	70/217 (32.3%)	26/143 (18.2%)	0.005
	Severe wasting**	62/160 (38.8%)	10/32 (31.3%)	72/192 (37.5%)	48/133 (36.1%)	0.887
	Mortality	38/178 (21.3%)	10/36 (27.8%)	48/214 (22.4%)	17/150 (11.3%)	0.01

Supplementary Table 7 | NTS serovars & co-morbidities in Kenyan & Malawian case samples. Distribution of NTS serovars and common NTS-associated co-morbidities in the Kenyan discovery, Kenyan replication and Malawian replication NTS cases. *P*-values represent comparisons of age (linear regression), or the proportions of a given clinical characteristic (χ^2 test), between study populations (Kenya total vs. Malawi replication).

*with available antisera.

**Severe wasting defined as weight-for-age z-scores <-3.

		rs13390936 genotype			
	Total (n=106)	AA (n=77)	AT (n=25)	TT (n=4)	*P*
Demographics					
Age, months (median, IQR)	16, 8-31	16, 8-33	14, 11-21	23, 11-49	0.4
Female (no., prop.)	50/106, 0.47	36/77, 0.47	12/25, 0.48	2/4, 0.5	0.88
Co-morbidity					
Malaria (no., prop.)	6/103, 0.06	3/75, 0.04	3/24, 0.13	0/4, 0	0.38
HIV (no., prop.)	43/104, 0.41	32/76, 0.42	10/24, 0.42	1/4, 0.25	0.65
Severe malnutrition (no., prop.)	24/98, 0.24	17/69, 0.25	6/25, 0.24	1/4, 0.25	0.97
Clinical features at admission					
Febrile (no., prop.)	83/99, 0.78	61/74, 0.82	20/22, 0.91	2/3, 0.67	0.78
Tachycardia* (no., prop.)	19/31, 0.61	16/26, 0.62	3/5, 0.6	ND	1
Tachypnoea* (no., prop.)	24/35, 0.69	18/28, 0.64	6/7, 0.86	ND	0.52
Respiratory distress** (no., prop.)	29/78, 0.37	19/54, 0.35	10/20, 0.5	0/4, 0	0.87
BCS<5 (no., prop.)	9/99, 0.09	8/72, 0.11	0/22, 0	1/4, 0.25	0.64
Outcome					
In-patient mortality (no., prop.)	14/106, 0.13	9/77, 0.12	5/25, 0.2	0/4, 0	0.73

Supplementary Table 8 | Demographics and clinical characteristics of Malawian NTS cases included in serum IFNγ experiment. Data is presented for the Malawian NTS cases (n = 106), for whom serum IFNγ levels during acute disease were correlated with genotype at rs13390936. *P*-values represent correlation of age (linear regression), or the proportions with a given clinical feature (χ^2 test for trend), with genotype at rs13390936. No, number; prop, proportion; ND, no data. *rates > 90th centile for age. **presence of any of intercostal/subcostal recession, nasal flare, tracheal tug, head bobbing.

			Kenya discovery		Kenya replication		Malawi replication	
			Cases	Controls	Cases	Controls	Cases	Controls
		Numbers*	180	2,677	38	1,336	150	339
		Median age in months (range)	15 (0-143)	BC	14 (0-52)	BC	16 (0-180)	18 (0-182)
		Females	47.2%	49.6%	50.0%	49.5%	45.3%	49.6%
Reported ethnicity	Kenya	Giriama	50.0%	45.8%	33.3%	40.0%		
		Chonyi	30.4%	37.1%	41.6%	44.1%		
		Kauma	7.6%	11.9%	8.3%	10.3%		
	Malawi	Chewa					20.7%	11.8%
		Yao					14.0%	19.2%
		Lomwe					28.7%	28.6%
		Ngoni					16.7%	24.8%

Supplementary Table 9 | NTS bacteraemia genetic association study sample demographics. Demographic data for cases and controls in Kenyan discovery, Kenyan replication and Malawian replication samples sets.

*Numbers included in final analysis. BC, birth cohort.

CONSORTIA MEMBERSHIP

The Kenyan Bacteraemia study group

Principal Investigators: Adrian V S Hill (Chair), Thomas N Williams, J Anthony G Scott, Stephen J Chapman

Key Personnel: Anna Rautanen, Tara C Mills, Kirk Rockett, Anne W Ndungu, Vivek Naranbhai, Alex W Macharia, Sophie Uyoga, Carolyne Ndila, Neema Mturi, Patricia Njuguna, Shebe Mohammed, James A Berkley, Isaiah Mwangi, Salim Mwarumba, Barnes S Kitsao, Brett S Lowe, Susan C Morpeth, Iqbal Khandwalla

The Kilifi DNA extraction Group: Alex W Macharia, Sophie Uyoga, Herbert Opi, Carolyne Ndila, Emily Nyatichi, Prophet Ingosi, Barnes Kitsao, Clement Lewa, Johnstone Makale, Adan Mohamed, Kenneth Magua, Mary Njoroge, Gideon Nyutu, Ruth Mwarabu, Metrine Tendwa and Thomas N Williams.

The Kilifi Bacteraemia Surveillance Group: Ismail Ahmed, Samuel Akech, Alexander Balo Makazi, Mohammed Bakari Hajj, Andrew Brent, Charles Chesaro, Hiza Dayo, Richard Idro, Patrick Kosgei, Kathryn Maitland, Kevin Marsh, Laura Mwalekwa, Shalton Mwaringa, Charles Newton, Mwanajuma Ngama, Allan Pamba, Norbert Peshu, Anna Seale, Alison Talbert and Thomas N Williams.

Wellcome Trust Case Control Consortium 2

Management Committee: Peter Donnelly (Chair), Ines Barroso (Deputy Chair), Jenefer M Blackwell, Elvira Bramon, Matthew A Brown, Juan P Casas, Aiden Corvin, Panos Deloukas, Audrey Duncanson, Janusz Jankowski, Hugh S Markus, Christopher G Mathew, Colin NA Palmer, Robert Plomin, Anna Rautanen, Stephen J Sawcer, Richard C Trembath, Ananth C Viswanathan, Nicholas W Wood

Data and Analysis Group: Chris C A Spencer, Gavin Band, Céline Bellenguez, Colin Freeman, Garrett Hellenthal, Eleni Giannoulatou, Matti Pirinen, Richard Pearson, Amy Strange, Zhan Su, Damjan Vukcevic, Peter Donnelly

DNA, Genotyping, Data QC and Informatics Group: Cordelia Langford, Sarah E Hunt, Sarah Edkins, Rhian Gwilliam, Hannah Blackburn, Suzannah J Bumpstead, Serge Dronov, Matthew Gillman, Emma Gray, Naomi Hammond, Alagurevathi Jayakumar, Owen T McCann, Jennifer Liddle, Simon C Potter, Radhi Ravindrarajah, Michelle Ricketts, Matthew Waller, Paul Weston, Sara Widaa, Pamela Whittaker, Ines Barroso, Panos Deloukas.

Publications Committee: Christopher G Mathew (Chair), Jenefer M Blackwell, Matthew A Brown, Aiden Corvin, Chris C A Spencer
